# Function-Wise Dual-Omics analysis for radiation pneumonitis prediction in lung cancer patients

**DOI:** 10.3389/fphar.2022.971849

**Published:** 2022-09-19

**Authors:** Bing Li, Ge Ren, Wei Guo, Jiang Zhang, Sai-Kit Lam, Xiaoli Zheng, Xinzhi Teng, Yunhan Wang, Yang Yang, Qinfu Dan, Lingguang Meng, Zongrui Ma, Chen Cheng, Hongyan Tao, Hongchang Lei, Jing Cai, Hong Ge

**Affiliations:** ^1^ Department of Radiation Oncology, The Affiliated Cancer Hospital of Zhengzhou University and Henan Cancer Hospital, Zhengzhou, China; ^2^ Department of Health Technology and Informatics, The Hong Kong Polytechnic University, Hong Kong SAR, China

**Keywords:** lung functional imaging, radiation pneumonitis, radiomics, dosiomics, radiotherapy

## Abstract

**Purpose:** This study investigates the impact of lung function on radiation pneumonitis prediction using a dual-omics analysis method.

**Methods:** We retrospectively collected data of 126 stage III lung cancer patients treated with chemo-radiotherapy using intensity-modulated radiotherapy, including pre-treatment planning CT images, radiotherapy dose distribution, and contours of organs and structures. Lung perfusion functional images were generated using a previously developed deep learning method. The whole lung (WL) volume was divided into function-wise lung (FWL) regions based on the lung perfusion functional images. A total of 5,474 radiomics features and 213 dose features (including dosiomics features and dose-volume histogram factors) were extracted from the FWL and WL regions, respectively. The radiomics features (R), dose features (D), and combined dual-omics features (RD) were used for the analysis in each lung region of WL and FWL, labeled as WL-R, WL-D, WL-RD, FWL-R, FWL-D, and FWL-RD. The feature selection was carried out using ANOVA, followed by a statistical F-test and Pearson correlation test. Thirty times train-test splits were used to evaluate the predictability of each group. The overall average area under the receiver operating characteristic curve (AUC), accuracy, precision, recall, and f1-score were calculated to assess the performance of each group.

**Results:** The FWL-RD achieved a significantly higher average AUC than the WL-RD group in the training (FWL-RD: 0.927 ± 0.031, WL-RD: 0.849 ± 0.064) and testing cohorts (FWL-RD: 0.885 ± 0.028, WL-RD: 0.762 ± 0.053, *p* < 0.001). When using radiomics features only, the FWL-R group yielded a better classification result than the model trained with WL-R features in the training (FWL-R: 0.919 ± 0.036, WL-R: 0.820 ± 0.052) and testing cohorts (FWL-R: 0.862 ± 0.028, WL-R: 0.750 ± 0.057, *p* < 0.001). The FWL-D group obtained an average AUC of 0.782 ± 0.032, obtaining a better classification performance than the WL-D feature-based model of 0.740 ± 0.028 in the training cohort, while no significant difference was observed in the testing cohort (FWL-D: 0.725 ± 0.064, WL-D: 0.710 ± 0.068, *p* = 0.54).

**Conclusion:** The dual-omics features from different lung functional regions can improve the prediction of radiation pneumonitis for lung cancer patients under IMRT treatment. This function-wise dual-omics analysis method holds great promise to improve the prediction of radiation pneumonitis for lung cancer patients.

## Introduction

Lung cancer is the leading cause of cancer-related death worldwide (Sung et al., 2021). Radiation therapy or radiotherapy (RT) is one of the golden-standard treatment techniques for patients with locally advanced non-small-cell lung cancer (NSCLC) (Kong et al., 2005; Chang et al., 2016). Study shows a higher radiation dose can achieve better tumor control and improve the treatment outcome (Kong et al., 2005). However, dose escalation of lung cancer is greatly limited by radiation-induced side effects, such as radiation pneumonitis (RP). RP may occur in up to 30% of lung RT patients and is lethal in 2% of them (Zhang et al., 2012; Kipritidis et al., 2015). Hence, predicting RP is highly desirable for better dose optimization and personalization to maximize the treatment outcome in lung cancer RT.

At present treatment planning of lung cancer RT, several dosimetric factors from the dose-volume histogram (DVH) were found to be associated with RP, such as V_5_, V_20_, and D_mean_ (Baisden et al., 2007; Barriger et al., 2012; Bongers et al., 2013; Palma et al., 2013; Cai et al., 2014; Pinnix et al., 2015). These parameters are commonly used as dose constraints in clinical plan evaluation (Ganti et al., 2021). Meanwhile, several prevalent models using DVH parameters, such as normal tissue complication probability (NTCP), were proposed to predict high risk RP patients (Begosh-Mayne et al., 2020; Wang et al., 2020). However, DVH parameters can only distinguish statistical one-dimensional dose information rather than characterizing the dose distribution heterogeneity. With the aid of the radiomics definition (Lambin et al., 2017), dosiomics features were calculated based on the three-dimensional dose distribution to describe the dose spatial information (Liang et al., 2019). Several studies also have demonstrated significantly superior models with the dosiomics feature compared to the DVH-based model or the NTCP model for predicting RP (Liang et al., 2019; Palma et al., 2019; Adachi et al., 2021). Meanwhile, CT-based radiomics features describe the statistical information, shaped, and textual characteristics in a certain volume. The dual-omics combines the radiomics and dose features and is able to further improves the prediction for RP (Adachi et al., 2021; Jiang et al., 2021; Puttanawarut et al., 2022). However, those radiomics or dose features utilized in current studies were calculated from the whole lung region, rather than considering the heterogeneity inside the lung, for example, the difference in high- and low- functional lung regions.

Lung function information has been proven to be associated with RP, which promises to improve the RP prediction accuracy (Bucknell et al., 2018; Lee et al., 2018; Weller et al., 2019; Bourbonne et al., 2020; O’Reilly et al., 2020). O’Reilly *et al.* demonstrated the RP prediction improvement using the DVH factor (V_20_) from three high functional lung regions and compared these biomarkers to the entire lung region (O’Reilly et al., 2020). Lee *et al.* evaluated the correlation between several DVH factors (V_5_, V_20_, and D_mean_) between the high functional lung region and the whole lung region, showing the potential of stratifying patients for pneumonitis prediction (Lee et al., 2018). Owen *et al.* demonstrated that irradiating to low functional lung region may increase radiation toxicity (Owen et al., 2021). Several studies also showed the potential of using dosimetry parameters based on functional lung images in predicting RP (Wang et al., 2012; Farr et al., 2015; Kimura et al., 2015; Xiao et al., 2018; Owen et al., 2021). However, these studies only explored the association between the dose factors and the RP without investigating the correlation between anatomical CT images and the RP. Besides, most studies focused on the dose features in the high functional lung region rather than the low functional lung region.

In this study, we developed a function-wise lung (FWL) analysis approach by integrating radiomics and dose features from both whole lung (WL) and FWL (including separated high- and low- functional lung) to predict RP for NSCLC patients. The radiomics features, dose features, and combined dual-omics features of each group were utilized for analysis. The feature selection metrics are the ANOVA followed by the statistical F-test and the Pearson correlation test. Thirty times train-test splits were used to evaluate the predictability of each group. The overall average area under the receiver operating characteristic curve (AUC), accuracy, precision, recall, and f1-score were calculated to assess the performance of each group.

## Materials and methods

### Data characteristics

The inclusion criteria are as follows: 1) diagnosed as primary locally-advanced lung cancer (stages IIIA/IIIC (AJCC 8th)); 2) having no distant metastasis; 3) treated with curative intensity-modulated radiotherapy (IMRT); 4) receiving contrast-enhanced CT for RT; 5) 18–70 years old. And the exclusion criteria are as follows:1) received chest RT or surgery or chemotherapy previously; 2) having previous chest malignancies; 3) received RT < 2 weeks; 4) incomplete RT treatment due to factors other than acute RP; 5) incomplete RT data.

**FIGURE 1:**
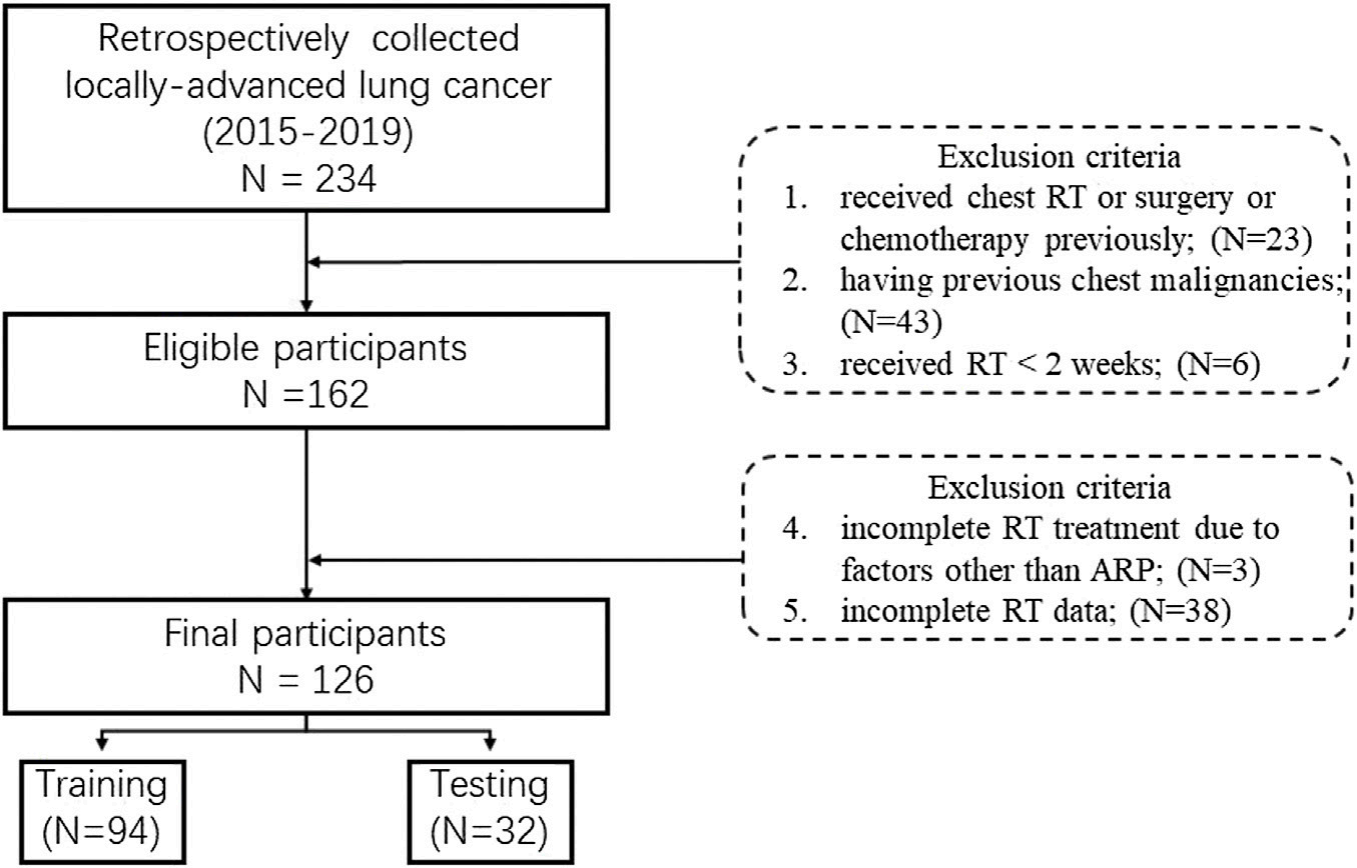
Flowchart of the inclusion and exclusion criteria.

The study was approved by the Institutional Review Board of the Affiliated Cancer Hospital of Zhengzhou University. Initially, a total of 162 pathological confirmed NSCLC patients staging IIIA/IIIC between 2015 and 2019 were retrospectively collected from the hospital. Considering the excluded criteria (shown in below), 126 cases were final enrolled in the study (Figure 1). All patients were treated by the 6 MV IMRT with a 50–70 Gy total prescription dose and 1.8–2.2 Gy fractional dose for 5 days per week. The radiation pneumonitis (RP) case was consecutively followed up at least 6 months after the first radiotherapy, and then graded with the Common Terminology Criteria for Adverse Events (CTCAE) V4.0. by one qualified imaging physician based on the CT scans. In this study, RP patients with grading ≥ 2 are defined as severe RP events because of dose escalation consideration.

### Image acquisition

Three types of image data were involved in this study, including planning CT images, three-dimension dose distribution images, and organs-at-risk (OAR) structures. All planning CT images were acquired from a 16-slice Brilliance Big Bore CT (Philips Medical System, Cleveland, OH, U.S.). The scanning parameters were as follows: scanning X-ray tube voltage = 120 kV, current = 321 mA, thickness = 3 mm, slice pixels = 512 × 512 and spacing = 1.152 mm × 1.152 mm. The scanning range was from the level of the cricoid cartilage to the lower border of the 12th thoracic vertebra covering the WL volume (Bradley et al., 2020). The 3D dose was calculated with a grid of 3 mm in the treatment planning system (TPS). The gross tumor volume was excluded from the lung volume with manually contouring by a qualified physician.

### Function-wise lung region

In this study, the functional images were generated using a previously developed deep learning neural network, which can translate the pulmonary anatomy information into functional information (Ren et al., 2021a; Ren et al., 2021b). In general, a 3D attention residual neural network was utilized to extract high level features from CT images and synthesize the perfusion images. This model was trained with CT and single-photon emission computerized tomography (SPECT) perfusion images of lung disease patients. This model used a 3D encoding-decoding structure to capture the hierarchical texture features of the input CT images with two attention modules to help focus on the defect regions, which is able to achieve a medium-to-high approximation with the ground truth SPECT perfusion images.

**FIGURE 2:**
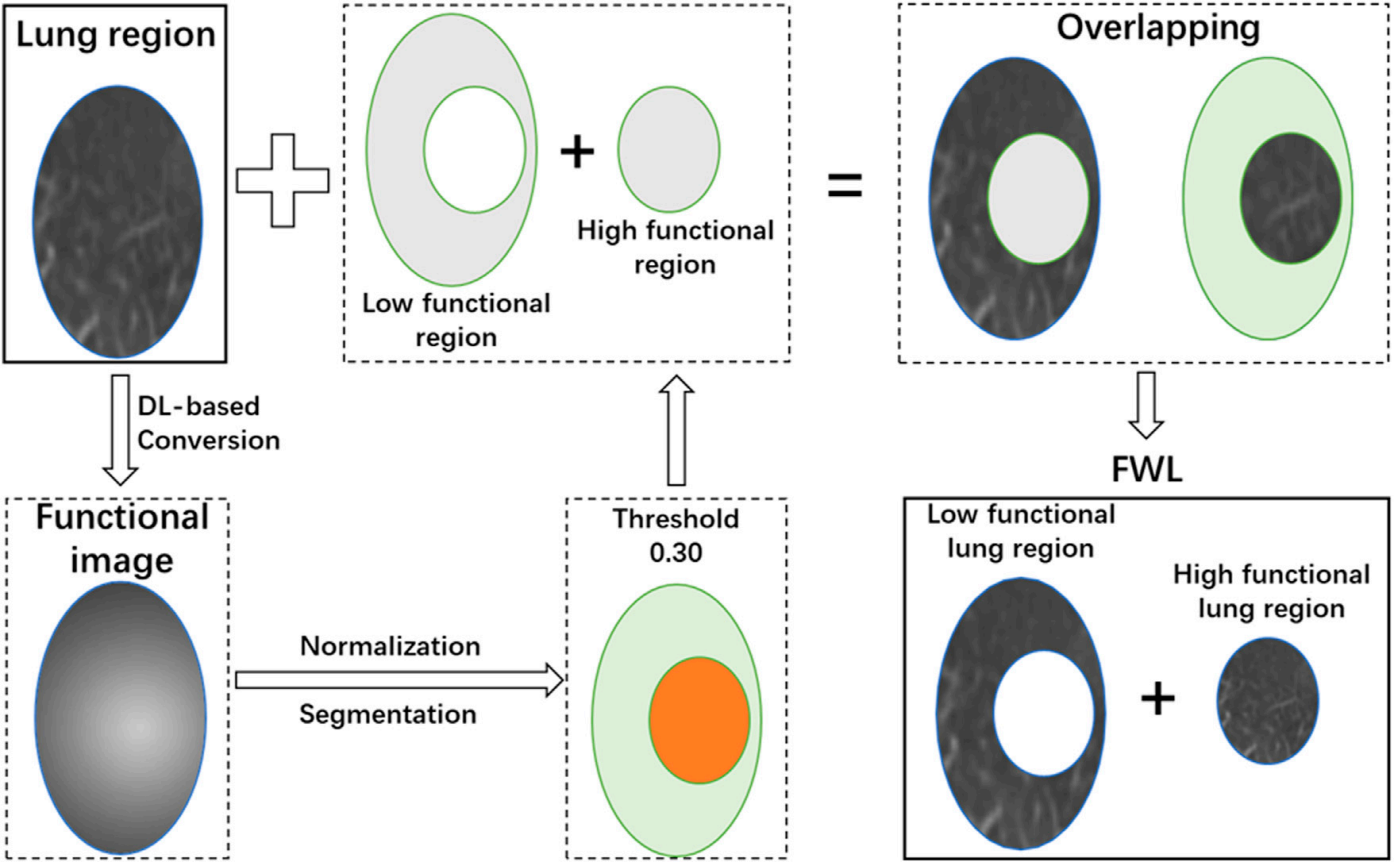
The scheme of the function-wise lung region generation.

After image synthesis, the functional image was normalized to the range of 0–1 by subtracting the minimum value and then divided by the maximum pixel value of function images. A threshold of 0.3 was used to divide the lung into high- and low- functional regions. Then the high- and low- functional lung regions were segmented on the CT image. This procedure is illustrated in Figure 2. After thresholding, the FWL was defined as the combination of the high- and low- functional lung regions. Besides, the WL region was also utilized as the basic comparison model.

### Feature extraction

In the study, radiomics features (Lambin et al., 2017) and dose features of the previous regions were extracted from the CT images and 3D dose distribution.

For radiomics features, the first-order and high-order radiomics features were extracted based on the original image and 11 filter-based images. The details of these radiomics features were described in the study (Lam et al., 2021). The only difference was the settings of bin counts, in the range of [20, 50, 100, 150, 200] and a total of 5,474 radiomics features were extracted from a region of interest (ROI).

The dose features can be categorized into three types: 1) scale-invariant 3D dose statistical moments (Pham et al., 2011), 2) DVH parameters (Marks et al., 2010; Faught et al., 2017), and 3) dosiomics features (Liang et al., 2019). The scale-invariant 3D dose statistical moments described the dose spatial distribution along three directions of anterior-posterior, medial-lateral, and craniocaudal (Pham et al., 2011). Except for the constant value of the order of [0, 0, 0], a total of 63 dose statistical moments were employed in the dose features. The DVH parameters consisted of Dx and Vx, where Dx is the dose larger than x% volume, and Vx is the volume larger than the x Gy or x% of the prescription dose. A total of 59 DVH parameters were included. The dosiomics features were radiomics features based on the image of 3D dose distribution. In the study, only the original image type was adopted in extracting dosiomics features. A number of 91 dosiomics features were extracted from the original 3D dose distribution in an ROI. Eventually, a total of 213 dose features were included.

In the study, two kinds of regions (WL and FWL) were used to extract features with a total of 5,687 features and 11,374 features, respectively.

### Feature selection

The feature dimension reduction is a crucial step to avoid model overfitting or underfitting. A combination of the F-test and the Pearson correlation test was utilized for the feature selection on the scikit-learn package in Python (version 1.0.1) (Pedregosa et al., 2011; Buitinck et al., 2013). Besides, the randomly under-sampling method was performed for comprehensively screening out the optimal feature group, which was described in the study (Yu et al., 2019; Lam et al., 2021).

**FIGURE 3:**
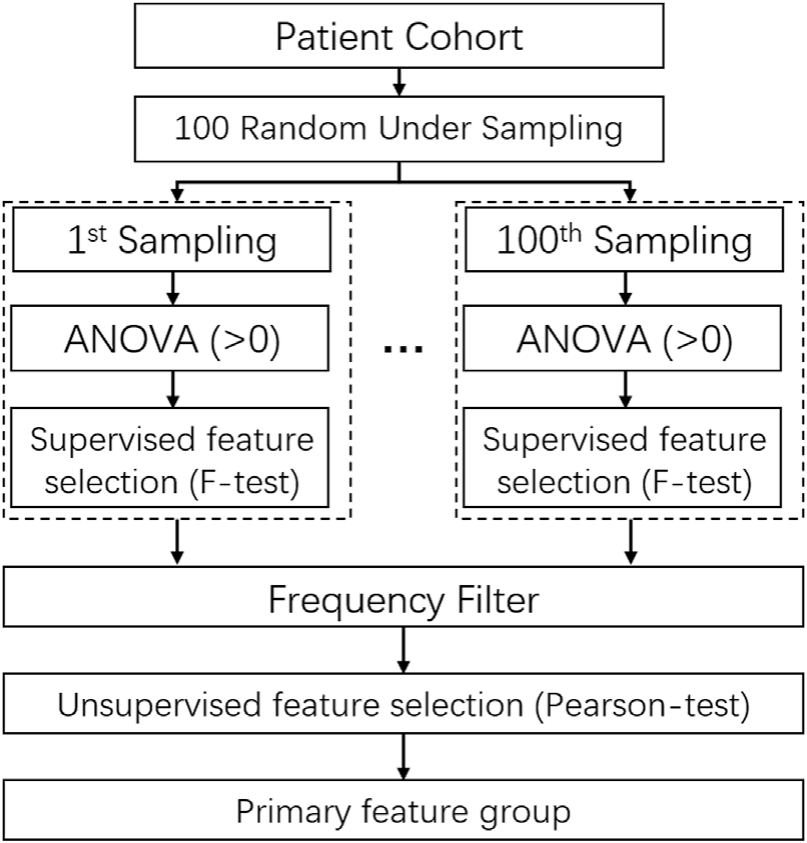
The scheme of randomly under-sampled feature selection method using unsupervised ans supervised feature selection algorithms.

The detail of feature selection is illustrated in Figure 3. 70% patients were randomly under-sampled from the whole patient cohorts by 100 times. At each sampling, all features with a variance of zero were filtered out to reduce the feature dimensions and the subsequent computational complexity. After that, the F-score of all features was calculated by combing the label data based on the F-test, and an F-score with a *p*-value smaller than 0.01 was marked as 1, otherwise as 0. Through 100 times sampling, a matric with 100×N (N: feature quantity with variance >0) was obtained. It is followed by the frequency filtering process to acquire more stable and robust features. Then, 10% of the quantity of all features or at least 40 features were reserved. Finally, the primary feature group was chosen by the Pearson correlation test with the threshold of coefficient of 0.5 as keeping the higher frequency one for two correlated features.

### Model construction and evaluation

In the study, two single-omics models, radiomics model (R) and dosiomics model (D), and the combined model (RD) were developed for WL and FWL regions separately, producing six models in total, labeled as WL-R, WL-D, WL-RD, FWL-R, FWL-D, and FWL-RD.

**FIGURE 4:**
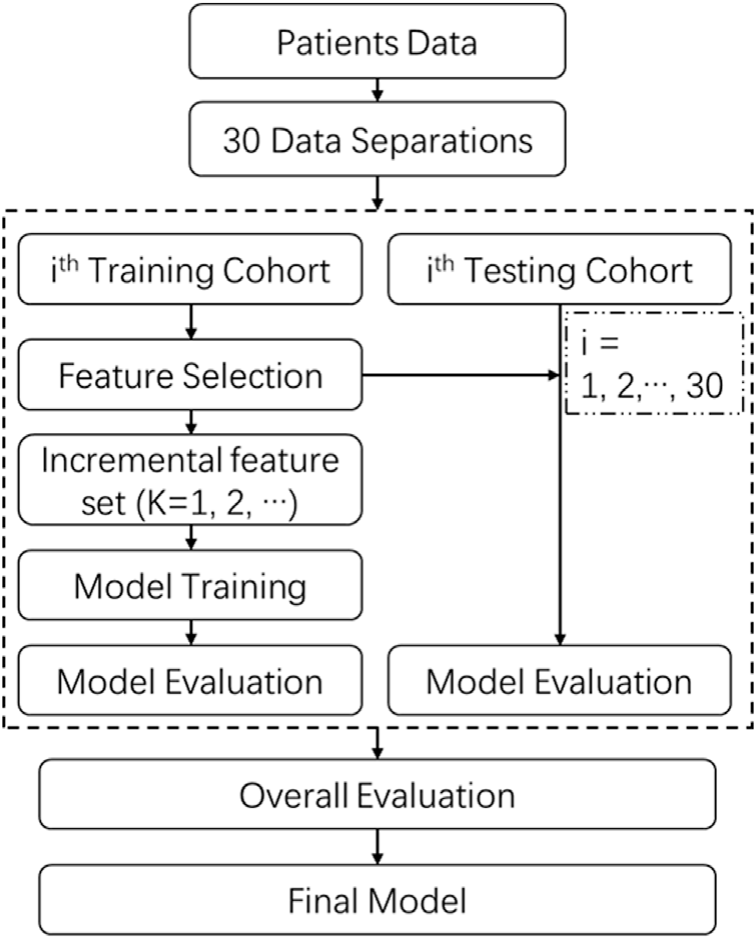
The scheme of model construction and evaluation.

The schematic diagram of the model development and evaluation is shown in Figure 4. All patient cohort was randomly divided into training and testing cohorts with a ratio of 3:1 across a repeat stratified splitting process of 30 times with different randomization, which simulated various patients’ data distributions to assess the model performance. At each split, training cohorts were sent to the procedure of feature selection, and the relevant primary feature group was obtained. Then, different feature combinations owning from one to all primary features were explored. The finally optimal feature group was determined by the maximum of the following overall average area under the receiver operating characteristic curve (ROC) curve (AUC) in the testing cohort. With the optimal feature combination, a classification regression algorithm of Ridge was utilized to develop a classification model using 10-fold cross-validation and hyper-parameters optimization search in the training cohort. The loss function for the Ridge classifier is $\underset{\omega }{\min}\|X_{\omega }-y{\|}_{2}^{2}+\alpha \|\omega {\|}_{2}^{2}$ , where α is complexity parameter with α>0 . After that, the model performance in the training and testing cohorts was performed by using a series of evaluation metrics, including accuracy, precision, recall, F1-score, and AUC. The average and the standard deviation (STD) were calculated in the training and testing cohorts by considering all splitting. The final model was evaluated by using the optimal feature group.

### Model comparison and statistic analysis

For each omics feature, the model performance using the corresponding omics features extracted from the function-wise lung regions was compared against the WL region in the training and testing cohorts using the five evaluation metrics. For each involved lung region, the model using dual-omics features was compared against the single-omics features in both training and testing cohorts using five evaluation metrics. Besides, net clinical benefits for all models were investigated using decision curve analyses (DCA) (Vickers and Elkin, 2006; Vickers et al., 2019). The DCA is a method to evaluate the clinical valuation of models overcoming the limitations of both traditional statistical metrics, such as discrimination and calibration.

The two-sided paired student *t*-test was utilized to compare the above-mentioned models with a group of features. On the other hand, the two-sided paired student *t*-test was also performed for the continuous clinical characteristics, while the Chi-square test was applied for the categorical variables. A *p*-value smaller than 0.05 was considered statistically significant. Except for the previously mentioned five evaluation metrics, the 95% confidence interval (CI) by the Delong method with (DeLong et al., 1988) 2000 times for all metrics was provided to access the ability to discriminate between severe RP cases and non-RP cases. Statistical analysis was performed with Python 3.7 and Pingouin 0.5.0 (Vallat, 2018).

## Results

### Patients characteristics

A total of 126 NSCLC patients were retrospectively involved in the study. The main characteristics of the patients are listed in Table 1. As shown in the table, 50.8% of patients (64 cases) developed the radiation pneumonitis with a grade ≥2. Except for the gender with a *p*-value of 0.04, the other clinical factors had no statistically significant difference between severe RP cases and non-RP cases.

**TABLE 1:** Patients’ characteristics.

Characteristics	Overall (126)
Gender	p=0.04
Male (N/%)	109/86.5%
Female(N/%)	17/13.5%
Age, median (range)	61 (29 -- 82) ( p=0.67 )
Pathology	p=0.46
SCC (N/%)	79/62.7%
ADC (N/%)	42/33.3%
Others (N/%)	5/4.0%
RT Dose, median (range)	60 (50–70) Gy ( p=0.94 )
Smoking	p=0.23
Activity or former (N/%)	97/77.0%
Never (N/%)	29/23.0%
Overall Stage	p=0.30
IIIA (N/%)	72/57.1%
IIIB (N/%)	37/29.4%
IIIC (N/%)	17/13.5%
Treatment method	p=0.97
SCRT (N/%)	83/65.9%
CCRT (N/%)	42/33.3%
RT (N/%)	1/0.8%
RP (N/%)	64/50.8%

SCC, squamous carcinoma cancer; ADC, adenocarcinoma cancer; SCRT, sequence chemoradiotherapy; CCRT, concomitant chemoradiotherapy.

### Optimal feature group

The final optimal features for six sets of WL-R, WL-D, WL-RD, FWL-R, FWL-D, and FWL-RD were listed in Supplementary Table S1 *.* The model performance with different feature numbers was plotted in Supplementary Figure S1 *.* A total of 39, 24, and 34 features were kept in the final optimal feature groups for FWL-R, FWL-D, and FWL-RD, respectively. The FWL-RD features consisted of 6 dosiomics and 28 radiomics features. For the region of WL, a total of 31, 4, and 29 features were utilized in the final optimal feature group for the R, D, and RD sets, respectively. The RD features consist of 6 dosiomics and 23 radiomics features. The feature number in the model of WL-R set was 35 with the maximum testing AUC. However, only 31 features were utilized in the final optimal feature group.

### Model performance

**FIGURE 5:**
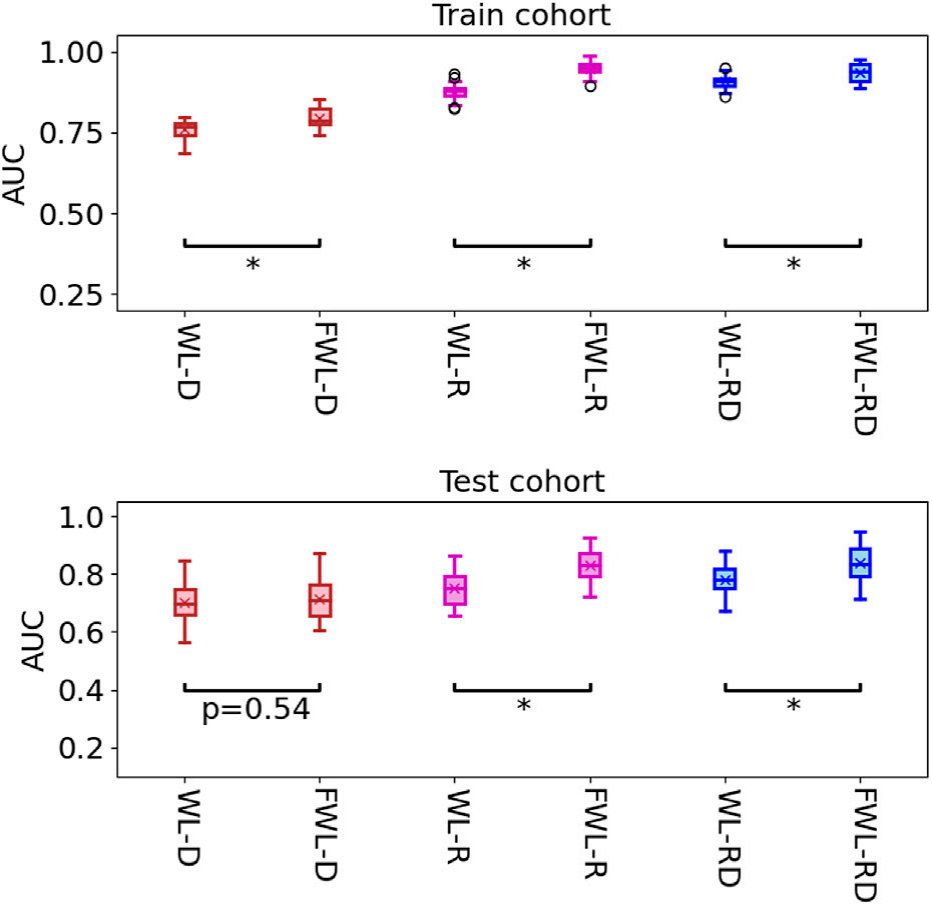
The comparison of model performance in the training and testing cohorts by using two region features of the whole lung and function-wise lung regions. The star means the p -value smaller 0.001 ( p<0.001 ).

Table 2 shows the average model performance for six feature sets of WL-D, WL-R, WL-RD, FWL-D, FWL-R, and FWL-RD in training and testing cohorts. Figure 5 shows model performance comparison between the WL and FWL models using each feature modality by considering 30 times data separations. For using dual-omics, the model using FWL-RD achieved significantly higher performance than the model using WL-RD in both training and testing cohorts, with an average AUC ± STD and 95% confidence interval of 0.927 ± 0.031 [0.917, 0.939]/0.849 ± 0.064 [0.823, 0.869] and 0.885 ± 0.028 [0.874, 0.893]/0.762 ± 0.053 [0.743, 0.781] ( p<0.001 ), respectively. For using radiomics, the model using FWL-R feature yielded a better classification result than the model using WL-R features both in the training and testing cohorts with AUC ± STD [95% CI] of 0.919 ± 0.036 [0.907, 0.933]/0.820 ± 0.052 [0.802, 0.838] and 0.862 ± 0.028 [0.851, 0.871]/0.750 ± 0.057 [0.730, 0.771] ( p<0.001 ), respectively. The FWL-D feature-based model performance with AUC ± STD [95% CI] of 0.782 ± 0.032 [0.771, 0.794] obtained a better classification performance than the WL-D feature-based model with AUC ± STD [95% CI] of 0.740 ± 0.028 [0.729, 0.750], however there were no significant difference in the testing cohorts with AUC ± STD [95% CI] of 0.725 ± 0.064 [0.703, 0.746] against to 0.710 ± 0.068 [0.686, 0.734] (*p* = 0.37).

**TABLE 2:** The average model performance in the training and testing cohorts using six feature sets of WL-D, WL-R, WL-RD, FWL-D, FWL-R, FWL-RD. The dark red color represents higher values.

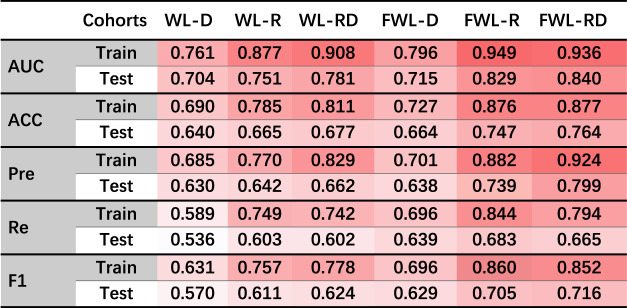

AUC, area under the receiver operator characteristic curve; ACC, accuracy; Pre, Precision; Re, Recall; F1, F1-score.

**FIGURE 6:**
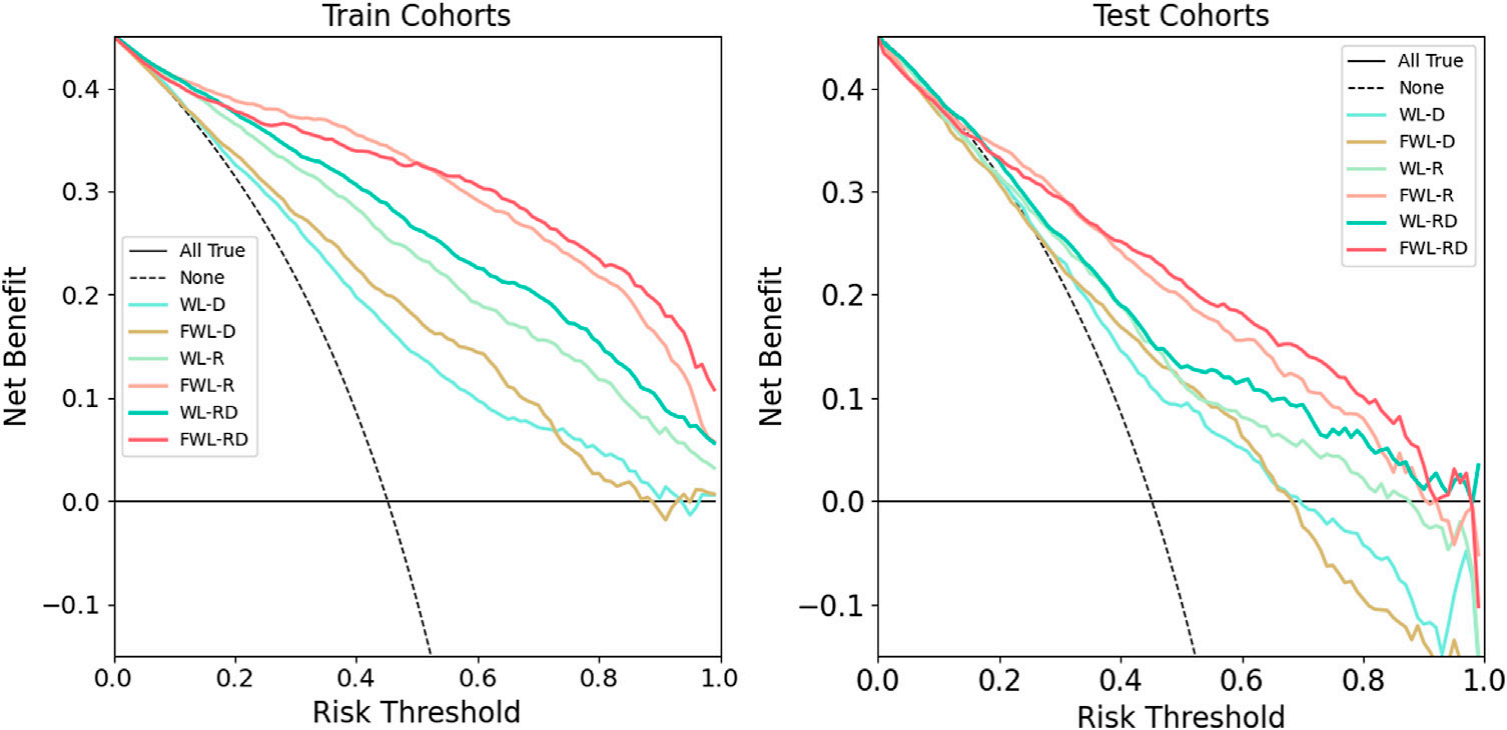
The comparison of clinical application values using decision curve analysis. The left and right plots showed the results of training and testing cohorts, respectively. The solid cyan line, solid brown line, solid light green line, solid pink line, solid green line, and solid red line represent the results of WL-D, FWL-D, WL-R, FWL-R, WL-RD, FWL-RD sets, respectively. The horizontal solid black line denotes that all patients didn’t suffer from RP. On the contrary, the dashed black line represents a condition that all patients occurred RP.

The decision curve analysis for all models is shown in Figure 6. The models using the FWL region’s feature performed a better clinical value than the models using WL region’s feature for both single and dual-omics. And, the model using FWL-RD achieved the highest overall net benefit across the majority of the range of reasonable threshold probabilities in both training and testing cohorts compared with the other feature group. ROC in the training and testing cohorts for all six models and their comparison in each feature modality and lung region were shown in Supplementary Figures S2, S3. For the best model with FWL-RD feature set, the weights of each final optimal features are displayed in Supplementary Table S3 *.*

## Discussion

In the study, we proposed an FWL sub-region generation method to benefit the prediction of acute radiation pneumonitis using pre-treatment imaging data. The predictability of each single omics and dual-omics (radiomics, dosiomics, and their combination) from the FWL were investigated and compared with the features from the WL region. As shown in Table 2 and Figure 6, the evaluation metrics and the decision curve analysis revealed that the FWL subregion generation method presented a significant prediction improvement in terms of radiomics and dual-omics features than using the WL region (*p < 0.001*), but not for dosiomics features.

For the models using FWL feature sets, the prediction accuracy has significant improvement as compared with the models using WL feature sets. This may suggest the features from both high and low functional lung regions have better prognostic power than the WL region. In FWL-R, FWL-D, and FWL-RD final features sets, there are 16, 7, and 15 features from the high functional regions, while they are 23, 2, and 19 for low functional regions. It should be noted that the high FWL dosiomics features played a more critical role in the FWL-D signatures. Several studies have showed the same conclusion for predicting RP when using the dose features from the high functional region (Yorke et al., 2002; Hunt et al., 2006; Wang et al., 2012; Hoover et al., 2014; Faught et al., 2017; Bucknell et al., 2018; Lee et al., 2018; O’Reilly et al., 2020). The low functional lung radiomics occupied a slightly more quantity than high FWL regions. The lower FWL’s radiomics signatures further lead to the more low FWL’s dual-omics signatures. It may imply that the heterogeneity of lung tissue, characterized by radiomics feature, presented both in high and low functional regions. The improvement for FWL-R to WL-R and FWL-RD to WL-RD may come from the smaller volume of region (high or low functional regions) than the WL region can benefit from extracting and distinguishing more heterogeneous radiomics features. Palm *et al.* (Palma et al., 2019) also found that the lower right lung has a significant correlation with radiation pneumonitis. In general, the lower functional region covers part of the lower right lung region. This may be one reason for the improvement by integrating the features from the low functional lung region.

For the FWL-RD signatures, except for one radiomics feature, the other radiomics signatures come from filtered CT images. The other radiomics signatures of FWL-RD are high-order features to describe gray level textural information of the lung region. For dosiomics signatures of FWL-RD, most of the signatures come from high-order features describing the dose distribution in the lung region or subregion. The selected final features are dominated by high-order omics features, which are also similar to previous studies (Hirose et al., 2020; Bourbonne et al., 2021; Jiang et al., 2021; Puttanawarut et al., 2022). None of DVH parameters (such as V_5_, V_20_, D_mean_) were included in our data study, which is inconsistent with the previous studies (Palma et al., 2013; Glick et al., 2018; Onishi et al., 2018).

In our dataset, the threshold of 0.3 only was adopted in dividing the lung into high and low functional lung regions. Previous studies report that the threshold can be different, ranging from 20% to the value of the maximum functional lung image pixel (Seppenwoolde et al., 2000; Kawakami et al., 2007; Lavrenkov et al., 2007; Ohno et al., 2011; Ding et al., 2018). Following their method, we have investigated the model performance using three omics features from the FWL regions using a list threshold from 0.2 to 0.8 with a step of 0.1, as shown in Supplementary Figure S4. Besides, we statistically analyzed the difference for the testing AUC between the threshold of 0.3 and the others by using the *t*-test, as shown in Supplementary Table S4. As shown in the figure and table, except for dosiomics, the threshold of 0.3 achieved a statistical higher classification result in testing cohorts for the majority of feature groups of radiomics and dual-omics (except for the threshold of 0.2, 0.6 and 0.8 in RD feature groups with *p* = 0.107, 0.054 and *p* = 0.343 respectively), which is consistent with a previous study (Seppenwoolde et al., 2000). Based on the previous observations, we determine the threshold of 0.3 as an optimal threshold by considering three kinds of features. For dosiomics, the threshold of 0.2 obtained a maximum AUC value, which agreed with the study (Ding et al., 2018). In addition, an optimal threshold only using the high functional lung region’s omics features was also assessed with the threshold list, as shown in Supplementary Figure S5. And the corresponding statistical analysis was shown in Supplementary Table S5. However, non-ignificant improvement (*p* > 0.05) was observed by comparing the high-functional lung regions to the whole lung region.

The current study still faces several limitations. First, the functional lung images generated by DL model may have uncertainties. Even though the DL-based approach can make the image acquisition convenient and less costly for patients, the uncertainty caused by the DL model can cause a discrepancy in high and low functional lung regions. This variance may finally affect the correlation relation between some omics features and the RP, thus changing final signature features. This proposed FWL approach should be verified using the real perfusion lung image. Second, all involved patients were treated by the IMRT technique. The other radiotherapy, such as volumetric modulated arc therapy and proton radiotherapy, should be investigated for our proposed FWL region method to further explore its feasibility and capability. Third, the unbalanced between the small sample cohort and a large number of features can induce overfitting both in training and testing cohorts (Hawkins, 2004). To minimize this effect, we adopted randomly under-sampling method in the feature selection to enhance the stability of final feature signatures. However, a large prospective cohort should be carried out to access the validation of our proposed FWL method. Finally, the reproducibility and stability of omics features were not validated against disturbance. Some studies have demonstrated that the reproducibility and stability of features can be affected by the dose calculation grid size and algorithm (Placidi et al., 2020), CT image acquisition, ROI segmentation (Zwanenburg et al., 2019), and time or volume change in 4D-CT (Larue et al., 2017; Lafata et al., 2018), etc. Therefore, it is important to validate the feature robustness before clinical application.

## Conclusion

In the study, we proposed an FWL approach to deeply explore the heterogeneous lung tissue and omics features and evaluated the approach in improvement of the prediction of the RP for lung cancer IMRT patients. The dual-omics features from different functional regions can improve the prediction of radiation pneumonitis for lung cancer patients under IMRT treatment. This function-wise dual-omics analysis method holds great promise to improve the prediction of radiation pneumonitis for lung cancer patients.

## Data Availability

The datasets generated for this study are available on request to the corresponding author.
