# Supplementary material for: Function-Wise Dual-Omics analysis for radiation pneumonitis prediction in lung cancer patients
Source: Front Pharmacol. 2022 Sep 19;13:971849. doi: 10.3389/fphar.2022.971849 (PMC9528994; doi:10.3389/fphar.2022.971849)
Supplement: Supplementary file 1 [file Table1.DOCX]

Supplementary Material

## Supplementary Tables

Supplementary Table 1 The selected features of three features sets (R, D, and RD) for both two regions of the whole lung region (WLR) and function-wise lung region (FWLR).

| Name | Features |
| --- | --- |
| FWL-R | Lung_higher_0.30_radiomics_wavelet-HLH_glszm_ZoneEntropy_50_binCount  Lung_higher_0.30_radiomics_wavelet-LHH_glszm_ZoneEntropy_100_binCount  Lung_lower_0.30_radiomics_wavelet-HLH_glszm_ZoneEntropy_50_binCount  Lung_lower_0.30_radiomics_wavelet-LLL_glszm_GrayLevelVariance_50_binCount  Lung_higher_0.30_radiomics_wavelet-LLH_gldm_DependenceVariance_20_binCount  Lung_lower_0.30_radiomics_wavelet-HLH_glcm_Imc1_20_binCount  Lung_lower_0.30_radiomics_log-sigma-1-0-mm-3D_glszm_ZoneEntropy_50_binCount  Lung_lower_0.30_radiomics_wavelet-HLH_glszm_ZoneEntropy_100_binCount  Lung_higher_0.30_radiomics_wavelet-LLH_glszm_ZoneEntropy_50_binCount  Lung_lower_0.30_radiomics_wavelet-HHL_glrlm_GrayLevelNonUniformityNormalized_20_binCount  Lung_lower_0.30_radiomics_log-sigma-6-0-mm-3D_glszm_LowGrayLevelZoneEmphasis_50_binCount  Lung_higher_0.30_radiomics_wavelet-LHH_glrlm_ShortRunLowGrayLevelEmphasis_200_binCount  Lung_lower_0.30_radiomics_wavelet-HHL_glcm_ClusterShade_20_binCount  Lung_higher_0.30_radiomics_wavelet-LHH_glrlm_LongRunHighGrayLevelEmphasis_150_binCount  Lung_higher_0.30_radiomics_wavelet-HHH_glcm_InverseVariance_50_binCount  Lung_lower_0.30_radiomics_wavelet-LLH_gldm_DependenceVariance_20_binCount  Lung_higher_0.30_radiomics_wavelet-HLH_glcm_Imc1_100_binCount  Lung_lower_0.30_radiomics_log-sigma-6-0-mm-3D_glszm_SizeZoneNonUniformityNormalized_20_binCoun  Lung_higher_0.30_radiomics_log-sigma-1-0-mm-3D_glszm_ZoneEntropy_20_binCount  Lung_higher_0.30_radiomics_wavelet-LHL_gldm_DependenceNonUniformity_20_binCount  Lung_lower_0.30_radiomics_wavelet-LLH_gldm_LargeDependenceLowGrayLevelEmphasis_20_binCount  Lung_higher_0.30_radiomics_wavelet-HLL_gldm_LargeDependenceLowGrayLevelEmphasis_20_binCount  Lung_lower_0.30_radiomics_wavelet-LHL_glszm_GrayLevelVariance_50_binCount  Lung_lower_0.30_radiomics_log-sigma-3-0-mm-3D_glcm_Imc2_20_binCount  Lung_lower_0.30_radiomics_log-sigma-1-0-mm-3D_ngtdm_Complexity_200_binCount  Lung_higher_0.30_radiomics_wavelet-HLH_gldm_DependenceNonUniformityNormalized_50_binCount  Lung_lower_0.30_radiomics_wavelet-HHH_glszm_ZoneEntropy_50_binCount  Lung_lower_0.30_radiomics_wavelet-LHL_firstorder_Kurtosis_20_binCount  Lung_lower_0.30_radiomics_wavelet-HLL_glszm_GrayLevelVariance_50_binCount  Lung_lower_0.30_radiomics_wavelet-HHH_glcm_Imc1_200_binCount  Lung_lower_0.30_radiomics_wavelet-LHH_glrlm_RunLengthNonUniformity_20_binCount  Lung_higher_0.30_radiomics_wavelet-HHL_glszm_ZoneEntropy_20_binCount  Lung_higher_0.30_radiomics_wavelet-HHH_glcm_Correlation_100_binCount  Lung_higher_0.30_radiomics_wavelet-LLL_glszm_SizeZoneNonUniformity_20_binCount  Lung_lower_0.30_radiomics_wavelet-LHL_firstorder_RootMeanSquared_200_binCount  Lung_higher_0.30_radiomics_wavelet-HHH_gldm_DependenceVariance_20_binCount  Lung_lower_0.30_radiomics_wavelet-HHH_glszm_SizeZoneNonUniformity_50_binCount  Lung_lower_0.30_radiomics_wavelet-HHH_glszm_ZoneEntropy_20_binCount  Lung_lower_0.30_radiomics_log-sigma-1-0-mm-3D_glszm_LowGrayLevelZoneEmphasis_50_binCount |
| FWL-D | Lung_higher_0.30_dosimetric_Lung_higher_0.30_original_ngtdm_Coarseness_1.00_binWidth  Lung_higher_0.30_dosimetric_Lung_higher_0.30_dose_moment_0_0_1  Lung_lower_0.30_dosimetric_Lung_lower_0.30_minimum_dose  Lung_higher_0.30_dosimetric_Lung_higher_0.30_original_gldm_DependenceVariance_1.00_binWidth  Lung_higher_0.30_dosimetric_Lung_higher_0.30_original_glrlm_RunEntropy_1.00_binWidth  Lung_higher_0.30_dosimetric_Lung_higher_0.30_dose_moment_0_1_2  Lung_lower_0.30_dosimetric_Lung_lower_0.30_dose_moment_2_1_3  Lung_higher_0.30_dosimetric_Lung_higher_0.30_dose_moment_3_0_2  Lung_higher_0.30_dosimetric_Lung_higher_0.30_original_glcm_Imc1_1.00_binWidth |
| FWL-RD | Lung_lower_0.30_radiomics_log-sigma-6-0-mm-3D_glszm_ZoneEntropy_50_binCount  Lung_higher_0.30_radiomics_wavelet-LHH_glszm_ZoneEntropy_100_binCount  Lung_lower_0.30_radiomics_log-sigma-6-0-mm-3D_glszm_ZoneEntropy_20_binCount  Lung_lower_0.30_radiomics_log-sigma-1-0-mm-3D_gldm_DependenceNonUniformityNormalized_20_binCou  Lung_higher_0.30_radiomics_wavelet-LHH_firstorder_Minimum_100_binCount  Lung_lower_0.30_radiomics_wavelet-HLH_glcm_Imc1_20_binCount  Lung_lower_0.30_dosiomics_Lung_lower_0.30_dose_moment_2_3_2  Lung_higher_0.30_radiomics_wavelet-HHH_glcm_InverseVariance_50_binCount  Lung_lower_0.30_radiomics_wavelet-HLH_glszm_ZoneEntropy_50_binCount  Lung_higher_0.30_radiomics_wavelet-HLH_glszm_ZoneEntropy_50_binCount  Lung_higher_0.30_dosiomics_Lung_higher_0.30_dose_moment_1_0_0  Lung_lower_0.30_radiomics_original_glszm_GrayLevelVariance_50_binCount  Lung_higher_0.30_radiomics_wavelet-LLH_glszm_ZoneEntropy_50_binCount  Lung_higher_0.30_radiomics_wavelet-LLH_gldm_DependenceVariance_20_binCount  Lung_lower_0.30_dosiomics_Lung_lower_0.30_original_firstorder_Minimum_1.00_binWidth  Lung_lower_0.30_radiomics_log-sigma-6-0-mm-3D_glszm_LowGrayLevelZoneEmphasis_50_binCount  Lung_higher_0.30_radiomics_log-sigma-1-0-mm-3D_glszm_ZoneEntropy_20_binCount  Lung_higher_0.30_radiomics_wavelet-HLH_glcm_Imc1_100_binCount  Lung_lower_0.30_radiomics_wavelet-HHL_glcm_ClusterShade_20_binCount  Lung_higher_0.30_dosiomics_Lung_higher_0.30_original_glrlm_ShortRunEmphasis_1.00_binWidth  Lung_higher_0.30_radiomics_original_glcm_InverseVariance_50_binCount  Lung_higher_0.30_dosiomics_Lung_higher_0.30_original_glrlm_RunEntropy_1.00_binWidth  Lung_lower_0.30_radiomics_wavelet-HHH_glszm_ZoneEntropy_20_binCount  Lung_lower_0.30_radiomics_wavelet-HLL_ngtdm_Busyness_20_binCount  Lung_lower_0.30_radiomics_log-sigma-3-0-mm-3D_glcm_Correlation_20_binCount  Lung_lower_0.30_radiomics_wavelet-LHL_glszm_GrayLevelVariance_50_binCount  Lung_higher_0.30_radiomics_wavelet-LHH_glrlm_ShortRunLowGrayLevelEmphasis_100_binCount  Lung_lower_0.30_radiomics_wavelet-HLH_glszm_ZoneEntropy_100_binCount  Lung_lower_0.30_radiomics_log-sigma-1-0-mm-3D_glszm_LowGrayLevelZoneEmphasis_50_binCount  Lung_lower_0.30_radiomics_wavelet-HLL_firstorder_Mean_150_binCount  Lung_higher_0.30_radiomics_wavelet-HHL_glszm_SizeZoneNonUniformity_50_binCount  Lung_lower_0.30_radiomics_log-sigma-6-0-mm-3D_glszm_ZoneEntropy_150_binCount  Lung_higher_0.30_dosiomics_Lung_higher_0.30_dose_moment_3_1_3  Lung_lower_0.30_radiomics_wavelet-LLH_glcm_Correlation_20_binCount |
| WL-R | Whole_Lung_radiomics_log-sigma-6-0-mm-3D_glszm_SmallAreaEmphasis_20_binCount  Whole_Lung_radiomics_log-sigma-6-0-mm-3D_glszm_ZoneEntropy_100_binCount  Whole_Lung_radiomics_wavelet-HLL_firstorder_Skewness_20_binCount  Whole_Lung_radiomics_log-sigma-3-0-mm-3D_glcm_Correlation_20_binCount  Whole_Lung_radiomics_wavelet-HLL_ngtdm_Busyness_20_binCount  Whole_Lung_radiomics_wavelet-HHL_glrlm_ShortRunLowGrayLevelEmphasis_20_binCount  Whole_Lung_radiomics_wavelet-LHL_ngtdm_Busyness_20_binCount  Whole_Lung_radiomics_original_glszm_ZoneEntropy_100_binCount  Whole_Lung_radiomics_wavelet-HLH_glcm_Imc1_20_binCount  Whole_Lung_radiomics_wavelet-HLH_glszm_ZoneEntropy_150_binCount  Whole_Lung_radiomics_log-sigma-3-0-mm-3D_glcm_Imc2_150_binCount  Whole_Lung_radiomics_wavelet-LHH_glcm_Imc1_50_binCount  Whole_Lung_radiomics_wavelet-HHL_glrlm_RunLengthNonUniformity_20_binCount  Whole_Lung_radiomics_wavelet-LLH_glrlm_RunEntropy_20_binCount  Whole_Lung_radiomics_wavelet-LLH_glszm_ZoneEntropy_20_binCount  Whole_Lung_radiomics_wavelet-LHH_glszm_SizeZoneNonUniformityNormalized_20_binCount  Whole_Lung_radiomics_log-sigma-6-0-mm-3D_glszm_ZoneEntropy_200_binCount  Whole_Lung_radiomics_wavelet-HLH_glrlm_LongRunLowGrayLevelEmphasis_200_binCount  Whole_Lung_radiomics_wavelet-LHH_glcm_InverseVariance_150_binCount  Whole_Lung_radiomics_wavelet-LHH_glszm_SizeZoneNonUniformity_20_binCount  Whole_Lung_radiomics_wavelet-HHL_gldm_DependenceEntropy_100_binCount  Whole_Lung_radiomics_wavelet-LHH_gldm_DependenceNonUniformityNormalized_50_binCount  Whole_Lung_radiomics_log-sigma-6-0-mm-3D_glszm_LowGrayLevelZoneEmphasis_100_binCount  Whole_Lung_radiomics_wavelet-HHL_firstorder_Mean_200_binCount  Whole_Lung_radiomics_wavelet-HHH_ngtdm_Busyness_50_binCount  Whole_Lung_radiomics_log-sigma-6-0-mm-3D_glszm_SizeZoneNonUniformity_20_binCount  Whole_Lung_radiomics_wavelet-LHL_glszm_GrayLevelVariance_50_binCount  Whole_Lung_radiomics_wavelet-LHH_glcm_Imc1_100_binCount  Whole_Lung_radiomics_log-sigma-6-0-mm-3D_glszm_LargeAreaLowGrayLevelEmphasis_150_binCount  Whole_Lung_radiomics_wavelet-HLL_glszm_GrayLevelNonUniformityNormalized_20_binCount  Whole_Lung_radiomics_wavelet-HHL_glrlm_GrayLevelVariance_20_binCount |
| WL-D | Whole_Lung_dosiomics_original_glrlm_RunEntropy_1.00_binWidth  Whole_Lung_dosiomics_original_firstorder_Minimum_1.00_binWidth  Whole_Lung_dosiomics_original_gldm_DependenceNonUniformity_1.00_binWidth  Whole_Lung_dosiomics_dose_moment_0_3_3 |
| WL-RD | Whole_Lung_radiomics_log-sigma-6-0-mm-3D_glszm_SmallAreaEmphasis_20_binCount  Whole_Lung_dosiomics_LungT_dose_moment_3_3_1  Whole_Lung_radiomics_log-sigma-6-0-mm-3D_glszm_ZoneEntropy_100_binCount  Whole_Lung_radiomics_wavelet-HLL_firstorder_Skewness_100_binCount  Whole_Lung_dosiomics_dose_moment_2_1_3  Whole_Lung_radiomics_log-sigma-3-0-mm-3D_glcm_Correlation_20_binCount  Whole_Lung_radiomics_wavelet-HLL_ngtdm_Busyness_20_binCount  Whole_Lung_dosiomics_minimum_dose  Whole_Lung_radiomics_wavelet-HHL_glrlm_ShortRunLowGrayLevelEmphasis_20_binCount  Whole_Lung_radiomics_wavelet-LHL_ngtdm_Busyness_20_binCount  Whole_Lung_radiomics_original_glszm_ZoneEntropy_100_binCount  Whole_Lung_radiomics_wavelet-HLH_glcm_Imc1_20_binCount  Whole_Lung_radiomics_wavelet-HLH_glszm_ZoneEntropy_150_binCount  Whole_Lung_radiomics_log-sigma-3-0-mm-3D_glcm_Imc2_150_binCount  Whole_Lung_radiomics_wavelet-LHH_glcm_Imc1_50_binCount  Whole_Lung_radiomics_wavelet-HHL_glrlm_RunLengthNonUniformity_20_binCount  Whole_Lung_radiomics_wavelet-HHL_glrlm_GrayLevelNonUniformityNormalized_20_binCount  Whole_Lung_radiomics_wavelet-LLH_glszm_ZoneEntropy_20_binCount  Whole_Lung_radiomics_wavelet-LHH_glszm_SizeZoneNonUniformityNormalized_20_binCount  Whole_Lung_radiomics_log-sigma-6-0-mm-3D_glszm_ZoneEntropy_200_binCount  Whole_Lung_dosiomics_original_glrlm_LongRunEmphasis_1.00_binWidth  Whole_Lung_dosiomics__original_firstorder_Energy_1.00_binWidth  Whole_Lung_radiomics_wavelet-HLH_glrlm_LongRunLowGrayLevelEmphasis_200_binCount  Whole_Lung_radiomics_wavelet-LHH_glcm_InverseVariance_150_binCount  Whole_Lung_radiomics_wavelet-LHH_glszm_SizeZoneNonUniformity_20_binCount  Whole_Lung_radiomics_wavelet-LHL_firstorder_Mean_20_binCount  Whole_Lung_radiomics_wavelet-HHL_gldm_DependenceEntropy_100_binCount  Whole_Lung_dosiomics__dose_moment_0_0_1  Whole_Lung_radiomics_wavelet-LHH_gldm_DependenceNonUniformityNormalized_50_binCount |

*MUAP: the product of MU and area, SF: shape feature, PSR: perimeter to the surface ratio, RF: rectangular factor, Q: quantity, T: summation, R: ratio.

Supplementary Table 2 The standard deviation of the model performance of the training and testing cohorts by using six feature sets of WL-D, WL-R, WL-RD, FWL-D, FWL-R, FWL-RD. Color represents the values that the greener, the lower.

|  | Cohorts | WL-D | WL-R | WL-RD | FWL-D | FWL-R | FWL-RD |
| --- | --- | --- | --- | --- | --- | --- | --- |
| AUC | **Train** | 0.028 | 0.024 | 0.019 | 0.031 | 0.021 | 0.025 |
|  | **Test** | 0.065 | 0.061 | 0.050 | 0.069 | 0.050 | 0.063 |
| ACC | **Train** | 0.034 | 0.034 | 0.033 | 0.034 | 0.034 | 0.029 |
|  | **Test** | 0.070 | 0.060 | 0.057 | 0.071 | 0.061 | 0.063 |
| Pre | **Train** | 0.050 | 0.038 | 0.052 | 0.048 | 0.054 | 0.042 |
|  | **Test** | 0.106 | 0.078 | 0.082 | 0.104 | 0.072 | 0.111 |
| Re | **Train** | 0.059 | 0.078 | 0.092 | 0.072 | 0.071 | 0.063 |
|  | **Test** | 0.121 | 0.144 | 0.113 | 0.123 | 0.119 | 0.105 |
| F1 | **Train** | 0.041 | 0.046 | 0.050 | 0.041 | 0.040 | 0.038 |
|  | **Test** | 0.089 | 0.093 | 0.078 | 0.077 | 0.087 | 0.070 |

**Supplementary Table 3** The weights of each final optimal FWL-RD features.

| FWL-RD Feature Name | Weight |
| --- | --- |
| Lung_lower_0.30_radiomics_log-sigma-6-0-mm-3D_glszm_ZoneEntropy_50_binCount | 0.019699 |
| Lung_higher_0.30_radiomics_wavelet-LHH_glszm_ZoneEntropy_100_binCount | 0.012781 |
| Lung_lower_0.30_radiomics_log-sigma-6-0-mm-3D_glszm_ZoneEntropy_20_binCount | 0.023591 |
| Lung_lower_0.30_radiomics_log-sigma-1-0-mm-3D_gldm_DependenceNonUniformityNormalized_20_binCou | 0.022149 |
| Lung_higher_0.30_radiomics_wavelet-LHH_firstorder_Minimum_100_binCount | 0.018932 |
| Lung_lower_0.30_radiomics_wavelet-HLH_glcm_Imc1_20_binCount | -0.01663 |
| Lung_lower_0.30_dosiomics_Lung_lower_0.30_dose_moment_2_3_2 | 0.021479 |
| Lung_higher_0.30_radiomics_wavelet-HHH_glcm_InverseVariance_50_binCount | -0.01857 |
| Lung_lower_0.30_radiomics_wavelet-HLH_glszm_ZoneEntropy_50_binCount | 0.009507 |
| Lung_higher_0.30_radiomics_wavelet-HLH_glszm_ZoneEntropy_50_binCount | 0.019774 |
| Lung_higher_0.30_dosiomics_Lung_higher_0.30_dose_moment_1_0_0 | -0.02226 |
| Lung_lower_0.30_radiomics_original_glszm_GrayLevelVariance_50_binCount | 0.015308 |
| Lung_higher_0.30_radiomics_wavelet-LLH_glszm_ZoneEntropy_50_binCount | -0.01419 |
| Lung_higher_0.30_radiomics_wavelet-LLH_gldm_DependenceVariance_20_binCount | 0.007327 |
| Lung_lower_0.30_dosiomics_Lung_lower_0.30_original_firstorder_Minimum_1.00_binWidth | 0.015861 |
| Lung_lower_0.30_radiomics_log-sigma-6-0-mm-3D_glszm_LowGrayLevelZoneEmphasis_50_binCount | -0.02229 |
| Lung_higher_0.30_radiomics_log-sigma-1-0-mm-3D_glszm_ZoneEntropy_20_binCount | -0.01173 |
| Lung_higher_0.30_radiomics_wavelet-HLH_glcm_Imc1_100_binCount | 0.012481 |
| Lung_lower_0.30_radiomics_wavelet-HHL_glcm_ClusterShade_20_binCount | 0.014854 |
| Lung_higher_0.30_dosiomics_Lung_higher_0.30_original_glrlm_ShortRunEmphasis_1.00_binWidth | 0.017977 |
| Lung_higher_0.30_radiomics_original_glcm_InverseVariance_50_binCount | -0.00759 |
| Lung_higher_0.30_dosiomics_Lung_higher_0.30_original_glrlm_RunEntropy_1.00_binWidth | -0.01094 |
| Lung_lower_0.30_radiomics_wavelet-HHH_glszm_ZoneEntropy_20_binCount | 0.016742 |
| Lung_lower_0.30_radiomics_wavelet-HLL_ngtdm_Busyness_20_binCount | 0.009533 |
| Lung_lower_0.30_radiomics_log-sigma-3-0-mm-3D_glcm_Correlation_20_binCount | 0.010243 |
| Lung_lower_0.30_radiomics_wavelet-LHL_glszm_GrayLevelVariance_50_binCount | -0.01697 |
| Lung_higher_0.30_radiomics_wavelet-LHH_glrlm_ShortRunLowGrayLevelEmphasis_100_binCount | 0.022114 |
| Lung_lower_0.30_radiomics_wavelet-HLH_glszm_ZoneEntropy_100_binCount | 0.011123 |
| Lung_lower_0.30_radiomics_log-sigma-1-0-mm-3D_glszm_LowGrayLevelZoneEmphasis_50_binCount | 0.009377 |
| Lung_lower_0.30_radiomics_wavelet-HLL_firstorder_Mean_150_binCount | 0.018274 |
| Lung_higher_0.30_radiomics_wavelet-HHL_glszm_SizeZoneNonUniformity_50_binCount | 0.016641 |
| Lung_lower_0.30_radiomics_log-sigma-6-0-mm-3D_glszm_ZoneEntropy_150_binCount | -0.01932 |
| Lung_higher_0.30_dosiomics_Lung_higher_0.30_dose_moment_3_1_3 | -0.00621 |
| Lung_lower_0.30_radiomics_wavelet-LLH_glcm_Correlation_20_binCount | -0.01406 |

Supplementary Table 4 The statistical analysis (p-value) for the testing AUC between the threshold of 0.3 and the others using the results of Supplementary Figure 4 for three kinds of features by using T-test method.

|  | WL | 0.2 | 0.3 | 0.4 | 0.5 | 0.6 | 0.7 | 0.8 |
| --- | --- | --- | --- | --- | --- | --- | --- | --- |
| D | 0.541 | 0.342 | 1.000 | 0.437 | 0.255 | 0.397 | 0.233 | 0.045 |
| R | 0.000 | 0.006 | 1.000 | 0.000 | 0.000 | 0.003 | 0.003 | 0.014 |
| RD | 0.000 | 0.107 | 1.000 | 0.001 | 0.015 | 0.055 | 0.002 | 0.343 |

Supplementary Table 5 The statistical analysis (p-value) for the testing AUC between the threshold of 0.3 and the others using the results of Supplementary Figure 5 for three kinds of features by using T-test method.

|  | WL | 0.2 | 0.3 | 0.4 | 0.5 | 0.6 | 0.7 | 0.8 |
| --- | --- | --- | --- | --- | --- | --- | --- | --- |
| D | 0.131 | 0.001 | 1.000 | 0.000 | 0.000 | 0.000 | 0.000 | 0.000 |
| R | 0.258 | 0.012 | 1.000 | 0.000 | 0.000 | 0.783 | 0.000 | 0.219 |
| RD | 0.519 | 0.001 | 1.000 | 0.000 | 0.000 | 0.010 | 0.008 | 0.014 |

## Supplementary Figures


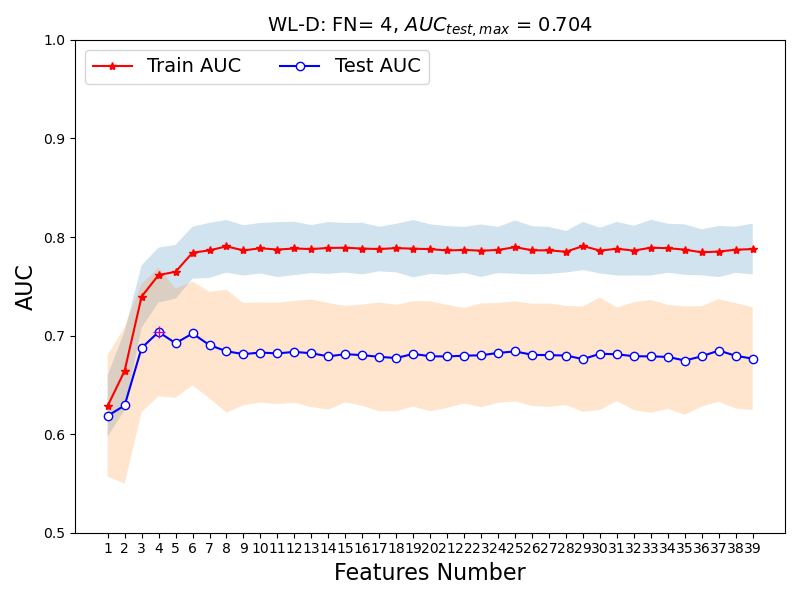

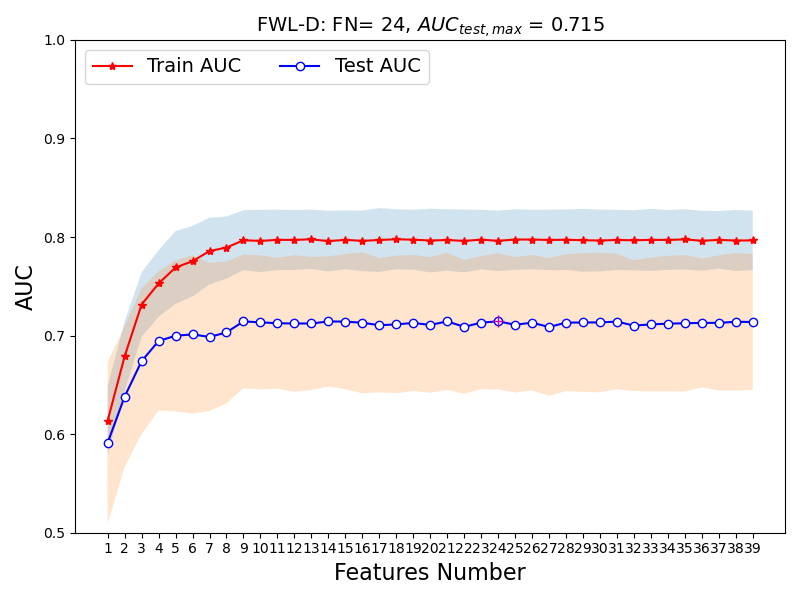

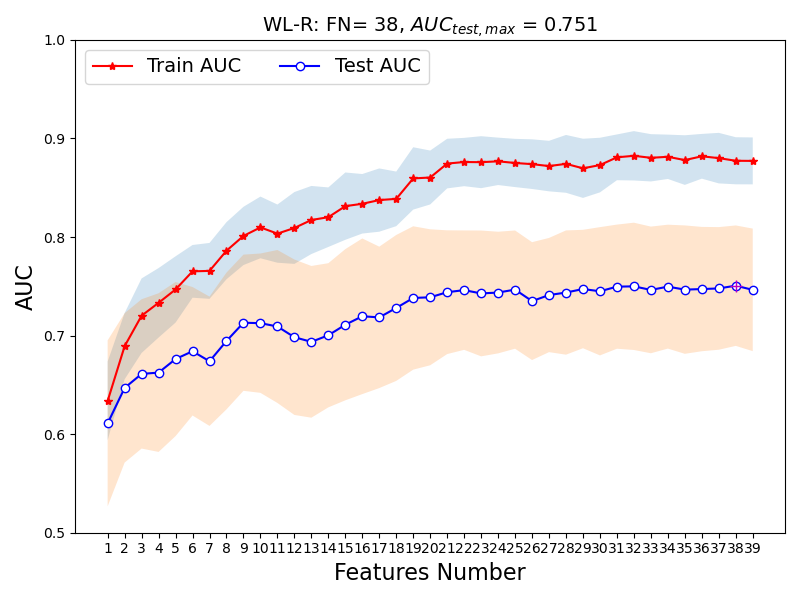

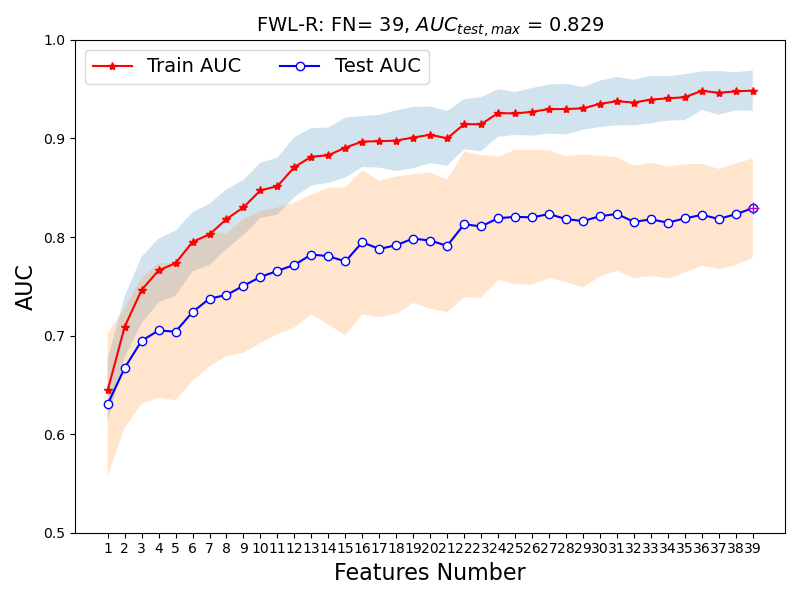

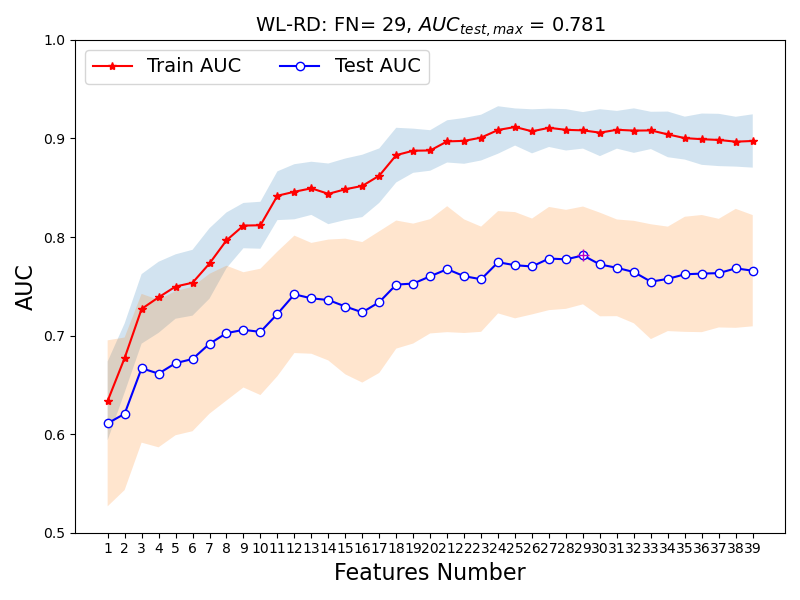

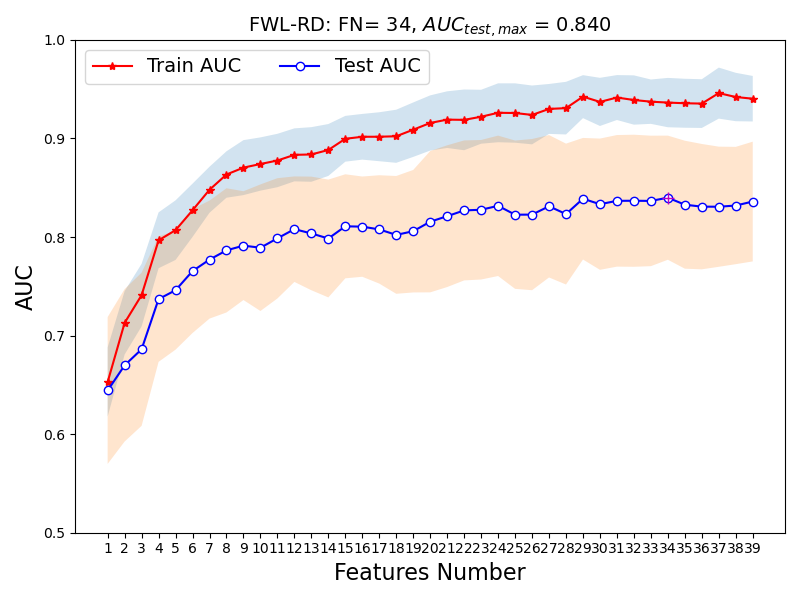


**Supplementary Figure 1** The model performance in the training and testing cohorts was varing with the feature number for using six feature groups. The first and second columns were the results of the models using features of WL region and FWL region, respectively. The red plus inside the blue circle denoted the maximum AUC in the testing cohorts for all models. The sharrow showed a standard deviation.


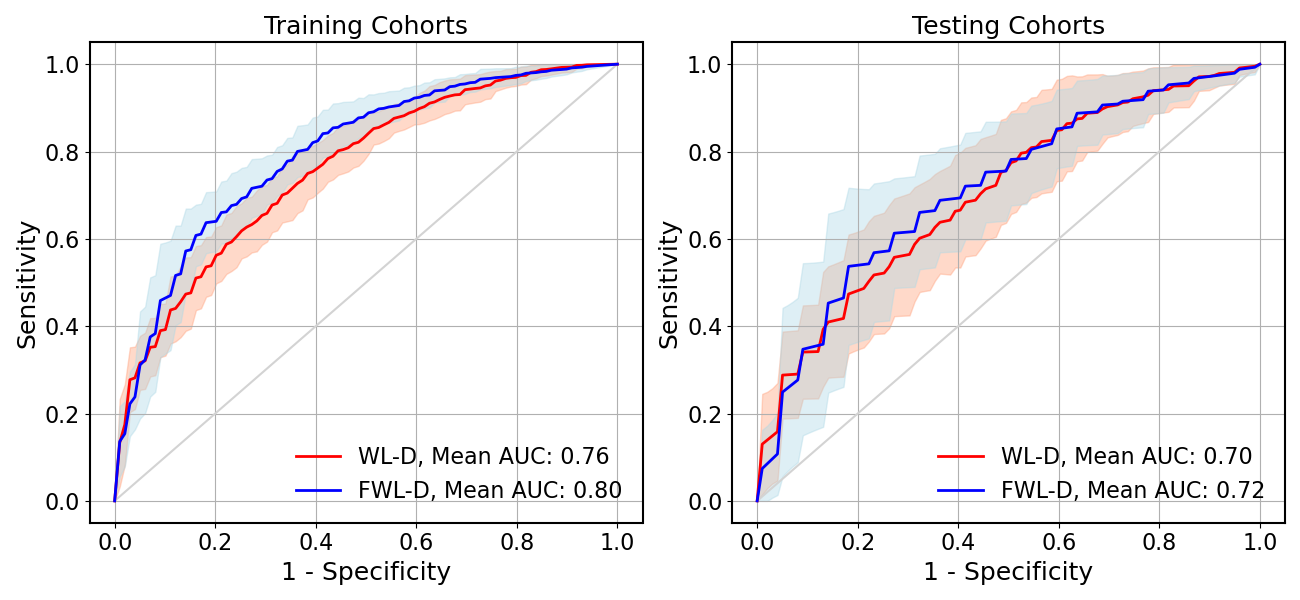

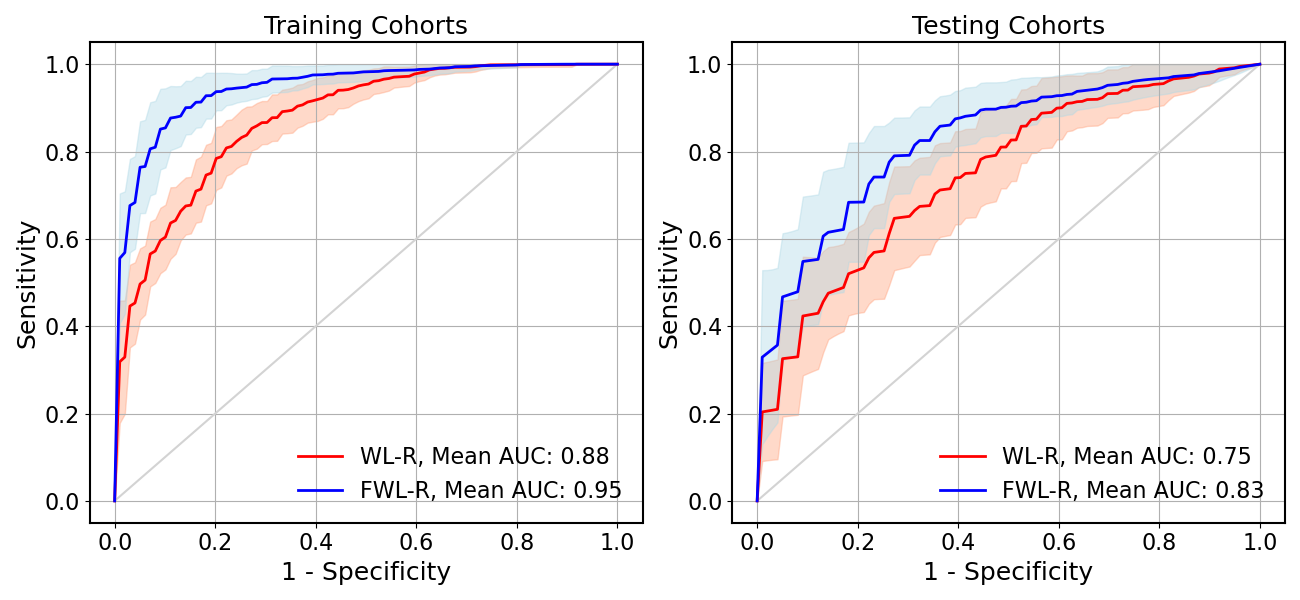

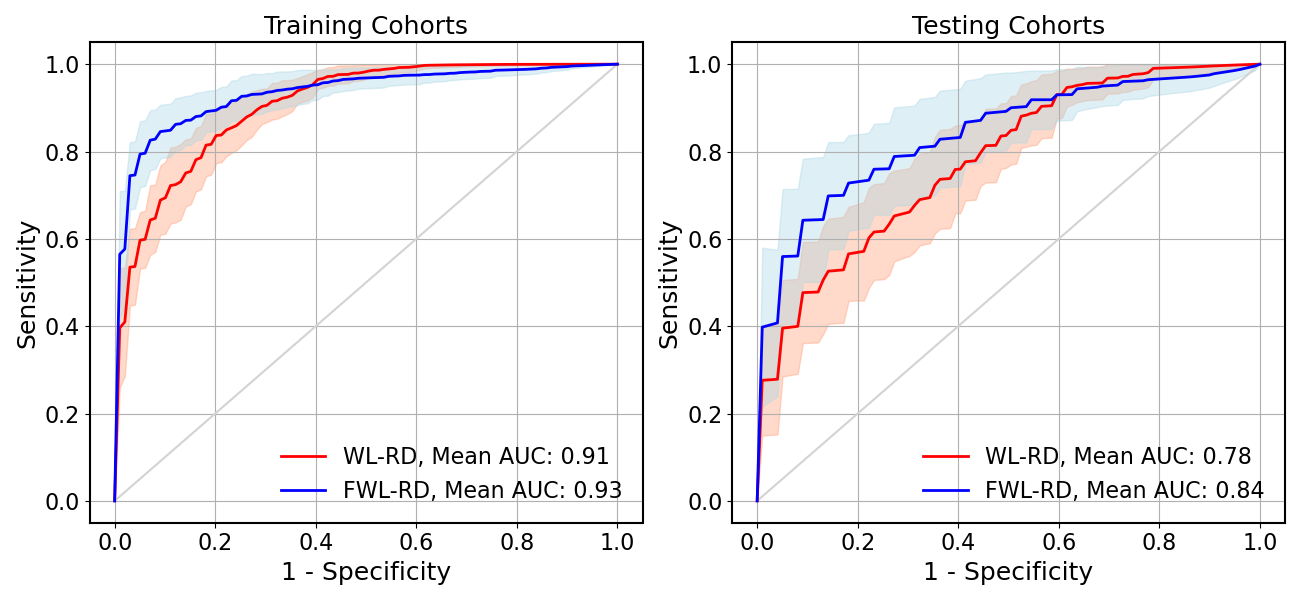


**Supplementary Figure 2** The ROC comparison in both training and testing cohorts for each dosiomics, radiomics, and multi-omics feature groups. The red and blue solided line denoted the features from the WL region and FWL region, respectively. The sharrow showed a standard deviation.


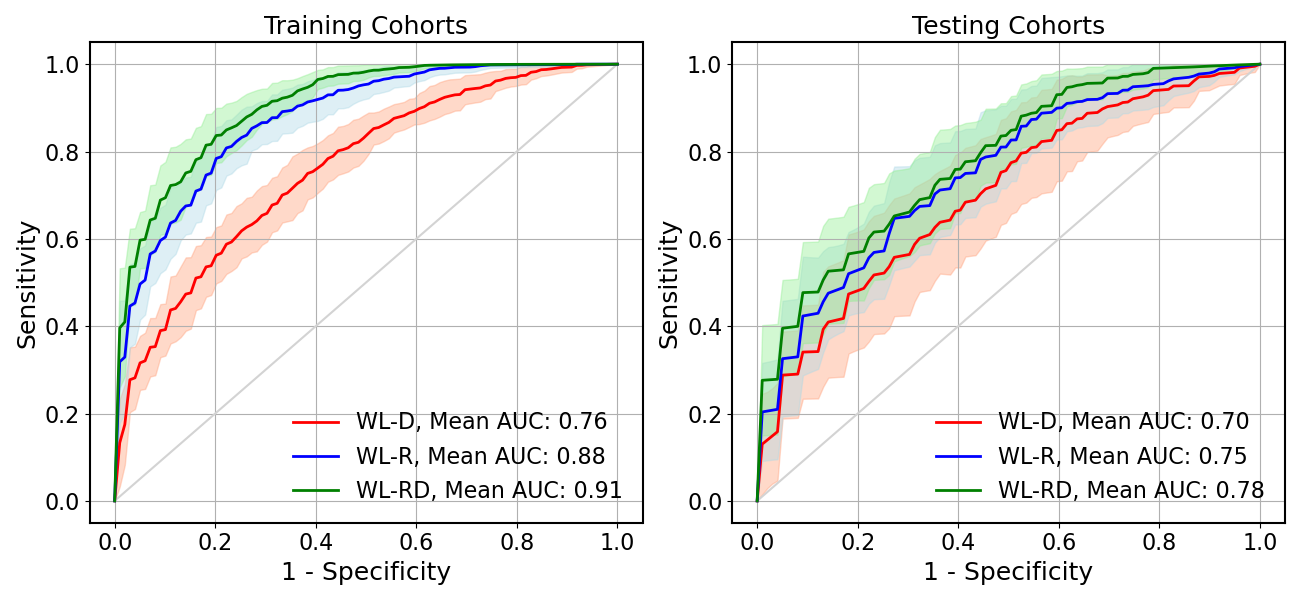

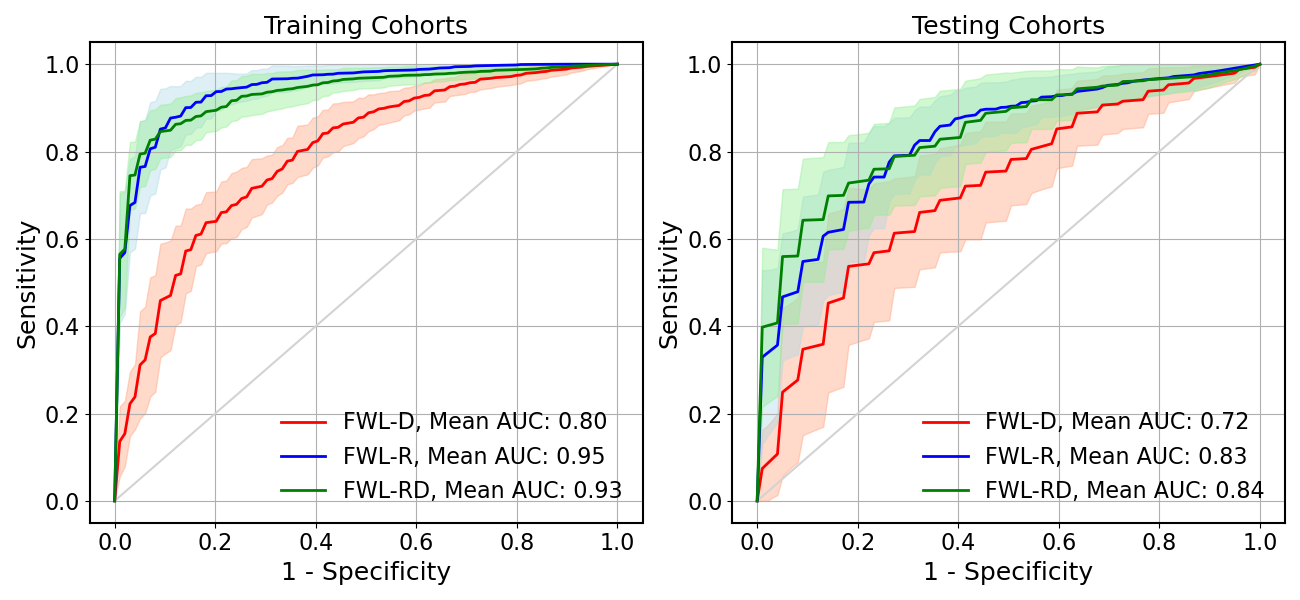


**Supplementary Figure 3** The ROC comparison in both training and testing cohorts is based on two lung regions of the whole lung (upper figure) and function-wise lung region (lower figure). The red, blue, and green solided line presented the dosiomics, radiomics, and multi-omics feature groups, respectively. The sharrow showed a standard deviation.


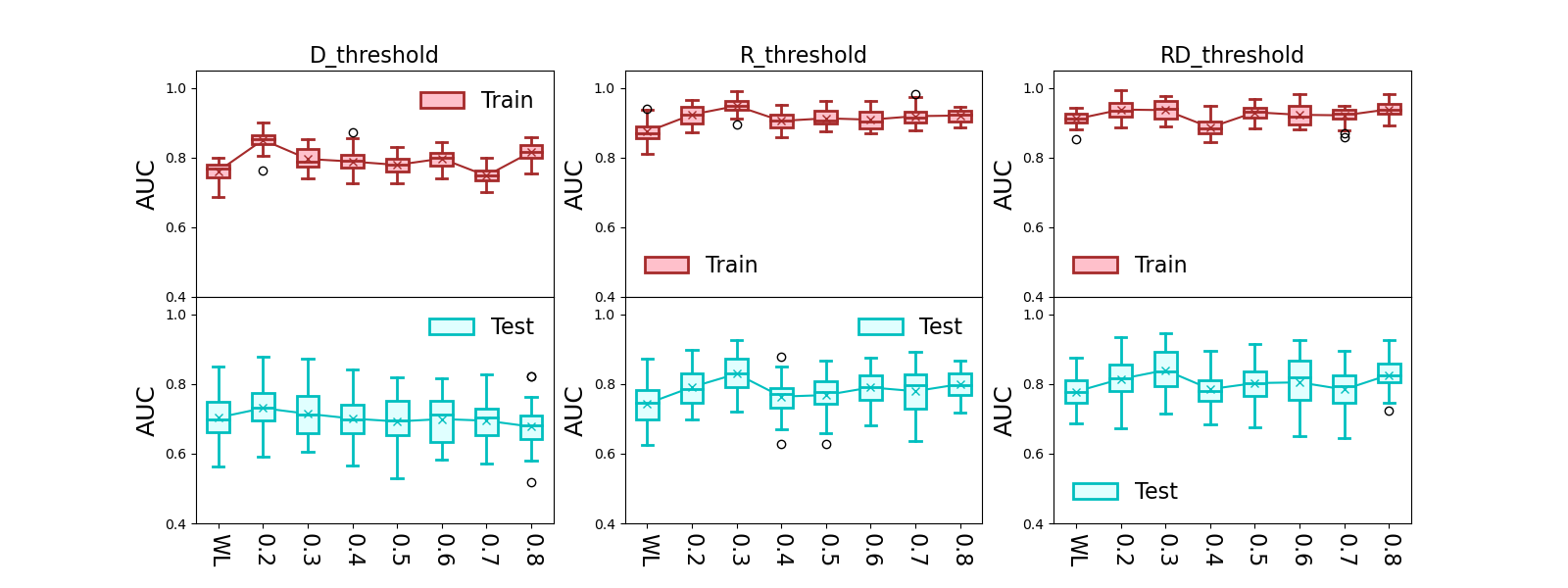


Supplementary Figure 4 Model performance using the three omics features from FWL regions was varied with the whole and seven threshold values [0.2, 0.3, 0.4, 0.5, 0.6, 0.7, 0.8].


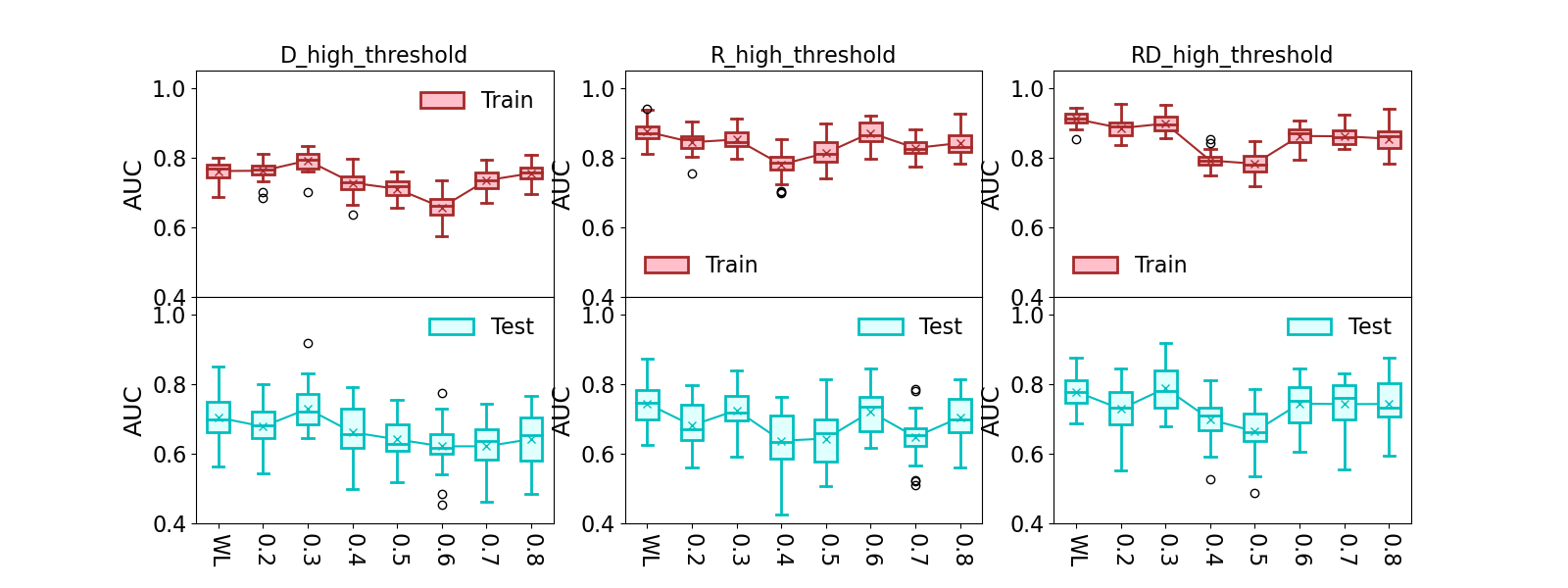


Supplementary Figure 5 Model performance using the three omics features from the high functional lung region was varied with the whole and seven threshold values [0.2, 0.3, 0.4, 0.5, 0.6, 0.7, 0.8].
